# Identifying spatial priorities for protecting ecosystem services

**DOI:** 10.12688/f1000research.1-17.v1

**Published:** 2012-09-27

**Authors:** Gary W Luck, Kai MA Chan, Carissa J Klien

**Affiliations:** 1 Institute for Land, Water and Society, Charles Sturt University, Albury, NSW, Australia; 2 Institute for Resources, Environment and Sustainability, University of British Columbia, Vancouver, British Columbia, Canada; 3 The Ecology Centre, University of Queensland, St. Lucia, Queensland, Australia

## Abstract

Priorities for protecting ecosystem services must be identified to ensure future human well-being. Approaches to broad-scale spatial prioritization of ecosystem services are becoming increasingly popular and are a vital precursor to identifying locations where further detailed analyses of the management of ecosystem services is required (e.g., examining trade-offs among management actions). Prioritization approaches often examine the spatial congruence between priorities for protecting ecosystem services and priorities for protecting biodiversity; therefore, the spatial prioritization method used is crucial because it will influence the alignment of service protection and conservation goals. While spatial prioritization of ecosystem services and prioritization for conservation share similarities, such as the need to document threats and costs, the former differs substantially from the latter owing to the requirement to measure the following components: supply of services; availability of human-derived alternatives to service provision; capacity to meet beneficiary demand; and site dependency in and scale of service delivery. We review studies that identify broad-scale spatial priorities for managing ecosystem services and demonstrate that researchers have used different approaches and included various measures for identifying priorities, and most studies do not consider all of the components listed above. We describe a conceptual framework for integrating each of these components into spatial prioritization of ecosystem services and illustrate our approach using a worked example for water provision. A fuller characterization of the biophysical and social context for ecosystem services that we call for should improve future prioritization and the identification of locations where ecosystem-service management is especially important or cost effective.

## Introduction

Ecosystem services (ES) are vital for human well-being ^1^. Much attention has been devoted to mapping and quantifying ES to achieve the dual goals of protecting biodiversity and human well-being. A growing number of broad-scale mapping studies aim to identify priority regions for conducting more localised place-based management of ES [e.g., ^2– 5^]. Place-based management requires intensive collection of detailed socio-economic and biophysical data, and close collaboration with stakeholders for effective decision making ^6, 7^. Given limited resources and information, and increasing threats to ecosystems, it is not possible to do these comprehensive analyses everywhere in a timely manner. We argue that there is currently an under-appreciated, but vital role for spatial prioritization of locations in which place-based management should occur so that attention is focussed on those locations where resource investment will yield the greatest return for human well-being. Indeed, data are deficient in most locations for informing comprehensive and accurate analyses of trade-offs in ES management, and spatial prioritization is a crucial precursor to attempting such trade-off analyses so that data mining efforts occur in the most critical locations. Moreover, prioritization is essential because much ES management is conducted by government or non-government organizations (NGOs) that could potentially operate in many places.

Given the important role that broad-scale prioritization can play in guiding decisions about where to conduct place-based ES management, a critical assessment of current prioritization approaches is warranted. Some schemes for identifying spatial priorities for managing ES are simple characterizations of biophysical processes and social demand, with little consideration of important information such as the availability of alternatives to ES for meeting human needs, threats to service provision, and the costs of management actions. Although fundamentally different to spatial prioritization for biodiversity conservation, spatial prioritization of ES may be guided by some of the key principles of the former. Spatial prioritization for conservation is well established and may be applied at coarse (e.g., biodiversity hotspots or priority ecoregions; ^8^) or fine scales, identifying locations or actions in locations that are relatively more important for protecting biodiversity than other actions or other locations ^9^. As with spatial prioritization of ES, spatial prioritization for conservation may help to identify locations where more detailed systematic conservation planning should be conducted, and is just one component of the planning process ^10, 11^.

Spatial prioritization of ES differs from spatial prioritization for conservation because ES are valued primarily for their worth to humans, can be transferable across space (may not need to be protected at a specific location), are sometimes substitutable by human engineering, and service beneficiaries define the success of management actions. Yet, as with spatial prioritization for conservation, spatial prioritization of ES can guide decsions about local-scale planning and inform the allocation of resources from management agencies (e.g., World Wildlife Fund; ^12^). Moreover, spatial prioritization for conservation is a useful starting framework for ES prioritization because the former is well entrenched in planning discourse ^13^ and yields valuable lessons for ES management ^14^.

Current approaches to identifying spatial priorities for managing ES apply different prioritization methods (see Table 1), and developing more consistent and comprehensive methods is an important goal for future prioritization studies. We review past approaches to spatial prioritization of ES, identifying key aspects that should be considered in future analyses. At appropriate places we discuss the relevance of spatial prioritization for biodiversity conservation to spatial prioritization of ES because certain aspects, such as accounting for costs and threats, are common to both. We then demonstrate the importance of these aspects through a conceptual framework for prioritization that outlines an approach for managing the most vital ES for the least cost where they are most needed ^15^. We illustrate the framework with a worked example using the ES of water provision. Egoh *et al.* ^14^ reviewed the extent to which ES were included in conservation assessments (≈ identifying spatial priorities). Our work differs from Egoh *et al.* by assessing *how* ES priorities have been identified and how methods for prioritization should be improved. It also complements discussions of other aspects of ES management such as how to operationalize ES on the ground ^16^, developing appropriate payments for services schemes (e.g., ^17, 18^) or how to manage service provision at specific sites [e.g., ^19, 20^].

**Table 1.** Studies identifying broad-scale spatial priorities for protecting ecosystem services (published from 2000–2011). Shown are the ecosystem services included in the study and how the authors expressed supply/benefits, demand, threats, costs or availability of alternatives to service provision. Blank cells represent a lack of information. A consistent typology for ecosystem services is not presented in the table because we have presented the ecosystem-service labels that were used in the original study.

Citation	Ecosystem services	Supply/Benefits	Demand	Threats	Costs	Alternatives
2 (see also Holland *et al.* 48 ^[note 1]^)	Carbon storage	Biophysical quantity ^[note 2]^				
	Agricultural value ^[note 3]^	Gross margin of crops and livestock ^[note 4]^				
	Recreation ^[note 5]^	# of visits ^[note 6]^				
53	Carbon sequestration	Biophysical quantity ^[note 7]^				
	Water quality	Amount of pollutants removed ^[note 8]^				
	Soil retention	Biophysical quantity ^[note 9]^				
	Water yield	Biophysical quantity				
	Pollination	Abundance of pollinators ^[note 10]^				
39	Carbon storage	Biophysical quantity	Target based ^[note 11]^		Area of planning unit ^[note 12]^	
	Flood control	Averted flood risk ^[note 13]^	Target based ^[note 14]^		Area of planning unit	
	Forage production ^[note 15]^	$ value ^[note 16]^	Target based ^[note 17]^		Sum of ‘development’ values ^[note 18]^	Implicit; integrated into benefit values
	Outdoor recreation ^[note 19]^	Biophysical quantity ^[note 20]^	12 days per person ^[note 21]^		Sum of ‘development’ values	
	Pollination ^[note 22]^	$ value ^[note 23]^	Target based ^[note 24]^		Area of planning unit	
	Water provision ^[note 25]^	Biophysical quantity	A fraction of actual use within each stratification unit ^[note 26]^		Area of planning unit	
4	Carbon storage	Biophysical quantity and $ value	Target-based and through $ value ^[note 27]^		Road-density proxy and services as added costs/benefits	
	Recreational angling	Biophysical quantity and $ value	Target-based and through $ value ^[note 27]^		Road-density proxy and services as added costs/benefits	
	Timber harvest	$ value (net: benefits – harvest cost)	Target-based and through $ value ^[note 27]^		Flat (costs included in $ value)	
47	Economic and cultural value of species ^[note 28]^	Binary categories ^[note 29]^		Threats from land use ^[note 30]^		
24, 54	Surface water supply	Biophysical quantity ^[note 31]^				
	Water flow regulation	Biophysical quantity ^[note 32]^				
	Soil retention	Erosion potential ^[note 33]^				
	Soil accumulation	Biophysical quantity ^[note 34]^				
	Carbon storage	Biophysical quantity				
3 (see also Egoh *et al.* 40; Reyers *et al.* 55)	Carbon storage	Biophysical quantity	Target based ^[note 35]^	Vegetation degradation ^[note 36]^	Conservation of planning unit and opportunity costs ^[note 37]^	
	Fodder provision ^[note 38]^	Biophysical quantity	Target based	Stocking rates ^[note 39]^	Conservation of planning unit and opportunity costs	
	Water recharge	Biophysical quantity ^[note 40]^	Target based		Conservation of planning unit and opportunity costs	
56 (see also Guo *et al.* 19)	Water retention ^[note 41]^	Biophysical quantity				
15	Water provision	Biophysical quantity ^[note 42]^	Supply relative to demand ^[note 43]^	Vegetation cover and loss ^[note 44]^	Proxy of costs per unit area ^[note 45]^	Capacity to pay for alternatives ^[note 46]^
	Flood mitigation	Biophysical quantity ^[note 47]^	Captured in measures of flood activity and HPD in watershed	Annual change in forest and woodland cover ^[note 48]^	Proxy of costs per unit area	Financial capacity to pay for alternatives (levee banks)
	Carbon storage	Biophysical quantity			Proxy of costs per unit area	
28	Carbon sequestration	Biophysical quantity		Land transformation ^[note 49]^		
	Economic value of marketable produce (e.g., timber, rice and non-timber forest produce)	Qualitative ranking ^[note 50]^	Inclusion of stakeholders ^[note 51]^			
	Renewal of soil fertility	Qualitative ranking ^[note 52]^				
34	Sustainable bushmeat consumption	$ value		Probability of conversion factors in threat	Opportunity costs ^[note 53]^	Market price of beef ^[note 54]^
	Sustainable timber harvest	$ value			Opportunity costs	
	Bio-prospecting ^[note 55]^	Willingness to pay			Opportunity costs	
	Existence value	Willingness to pay			Opportunity costs	
	Carbon storage	$ value		Deforestation ^[note 56]^	Opportunity costs	
26	Carbon sequestration	Biophysical quantity ^[note 57]^			Area constraint ^[note 58]^	
	Carbon storage	Biophysical quantity			Area constraint	
	Grassland production of livestock	Biophysical quantity ^[note 59]^	Variation in human population density ^[note 60]^		Area constraint	
	Water provision	Biophysical quantity ^[note 61]^			Area constraint	
30 ^[note 62]^	Water quality	Biophysical quantity		Landscape change ^[note 63]^		
	Storm peak mitigation	Biophysical quantity		Landscape change		
	Soil conservation ^[note 64]^	Biophysical quantity		Landscape change		
	Carbon sequestration	Biophysical quantity and social value (in $)		Landscape change		
57	Water supply	Biophysical quantity ^[note 65]^	Identified beneficiaries ^[note 66]^			
	Grazing provision	Biophysical quantity ^[note 67]^				
	Tourism	Distance-based aesthetics ^[note 68]^				
58	Soil and water conservation ^[note 69]^	Landslide, flood and drought prevention ^7[note 70]^		Deforestation potential ^[note 71]^		
55	Forage production for livestock	Biophysical quantity ^[note 72]^		Land-cover change ^[note 73]^		
	Carbon storage	Biophysical quantity		Land-cover change		
	Erosion control	Vulnerability to erosion ^[note 74]^		Land-cover change		
	Freshwater flow and quality regulation	Biophysical quantity ^[note 75]^		Land-cover change		
	Tourism	Distance-based aesthetics ^[note 76]^		Land-cover change		
31 ^[note 77]^	Hydrological services	Biophysical quantity ^[note 78]^		Human pressure index related to key biodiversity areas ^[note 79]^		
59	Carbon storage	Biophysical quantity ^[note 80]^				
29	Various ^[note 81]^	$ value ^[note 82]^		Land transformation ^[note 83]^		
60	Various ^[note 84]^	$ value		Vulnerability of biodiversity ^[note 85]^		
41 (see also Bohensky *et al.* 43).	Freshwater provision	Biophysical quantity ^[note 86]^	Water use and access ^[note 87]^			
	Food provision	Biophysical quantity ^[note 88]^	Dietary intake ^[note 89]^			
	Wood fuel	Biophysical quantity (local production)	Local harvest rate			
61	Carbon storage	Biophysical quantity		Deforestation rates and cover of protected areas	Opportunity costs	
18	Carbon storage	Biophysical quantity		Probability of deforestation	Opportunity costs ^[note 90]^	
	Water quality	Proxy ^[note 91]^	Estimated downstream users ^[note 92]^	Probability of deforestation		

Table 1 : Notes

1. Holland *et al.* ^48^ used four indicators of river status – environmental quality index, taxon richness, habitat quality assessment and habitat modification index – to represent the capacity of river systems and catchments to provide freshwater ecosystem services. The authors argue that changes in the value of these indices reflect changes in the capacity of river systems to provide services such as maintaining water quality, controlling sedimentation and erosion, mitigating floods, cycling nutrients, and filtering pollutants.

2. Carbon stored in soils and vegetation. The authors conducted analyses at different grain sizes (4 km ^2^ and 100 km ^2^) and different spatial extents (Britain/England and 100 × 100 km squares across Britain) and examined variation across regions within Britain.

3. Annual income.

4. The gross margin is the value of outputs minus variable costs and subsidy payments.

5. Recreational use of the countryside.

6. The number of day leisure visits as a measure of the recreational value of particular rural locations (this measure could be interpreted as the demand for recreational services).

7. Amount of carbon sequestered each year.

8. Nitrogen and phosphorus removed in particular landscapes.

9. Capacity of land to retain sediment.

10. Combining information on nest sites, floral resources and bee flight ranges to estimate pollinator abundance and likely visitation to agricultural areas.

11. The authors set targets to address the issue of demand (e.g., capturing 50% of total carbon stored in an ecoregion).

12. Costs are represented by the suitability of areas for conservation based on numerical values that reflect the degree of impediments to conservation success. For carbon storage it is a flat cost; the area of the planning unit.

13. Averted risk of extreme floods.

14. The fraction of total flood control value, as a function of the number of housing units in the floodplain.

15. Production of forage for grazing rangeland stock.

16. Dollar value of forage production.

17. The target was 75% of forage production value.

18. The sum of weighted values associated with developed land, agriculture, road density and length of human-induced patch edges.

19. Provision of recreation opportunities.

20. Quantity of suitable habitat in addition to accessibility issues and rights to access.

21. A baseline target (assumed minimum requirement) of 12 days of outdoor recreation per person per year.

22. Crop pollination by natural pollinators.

23. The dollar value of agricultural crops benefitting from pollination.

24. 75% of feature value across the ecoregion.

25. The supply of fresh water.

26. 40% of total freshwater use.

27. The authors pursued two approaches, a target-based approach and incorporating ecosystem services as extra costs or benefits in the cost layer.

28. This is a species-based approach so the priorities are based on species and their distribution across the landscape.

29. For example, positive or negative economic value.

30. The magnitude of threats affecting each species based on major land uses. The loss of a species is equivalent to the loss of the service(s) that species provides.

31. Median annual simulated run-off.

32. Groundwater contribution to surface run-off.

33. Hotspots mapped as areas with severe erosion potential and vegetation and litter cover of at least 70% where maintaining the cover is essential to prevent erosion.

34. Soil depth and leaf litter.

35. The authors assessed various scenarios for capturing ecosystem services based on incidental protection through the conservation of biodiversity or the inclusion of spatially explicit data on service distribution using Marxan. In Egoh *et al.* ^40^, the authors set different target thresholds for capturing certain percentages of service provision for surface water supply, water flow regulation, carbon storage, soil retention and soil accumulation.

36. The authors estimated the amount of each ecosystem service provided by vegetation types under intact and degraded conditions. Measuring the difference between the two is indicative of the threat of degradation to service provision.

37. The cost of conserving a planning unit was equivalent to the value of irrigated cropping or grazing. The opportunity costs of conservation were addressed in terms of lost production. The authors included spatial variability in costs because values are per planning unit. In Egoh *et al.* ^40^, catchment area is used as a cost layer (larger areas = greater cost).

38. By natural vegetation.

39. The authors examined the relationship between fodder provision and stocking rates to determine the stocking rates that can be implemented without degrading the environment (i.e., sustainable stocking rates). Hence, over-stocking is considered implicitly as a threat to vegetation condition.

40. Groundwater recharge.

41. For example, for flood mitigation. The authors also examined opportunities for service enhancement.

42. Incorporating the density of people who rely on the service (beneficiaries) as density per watershed, and the water–production efficiency as water supply divided by area of watershed.

43. Water supply relative to demand adjusted for the need to redistribute supply within watersheds. Watersheds were supply does not (or only just) meets demand were prioritized.

44. Amount of vegetation cover and rate of vegetation loss with mid-range values designated as priorities.

45. A proxy was used representing resource and maintenance costs (e.g., land acquisition, infrastructure and labour) and considering watershed-level measures of income, population size and area.

46. Financial capacity to pay for alternatives to service provision such as dams and filtration plants.

47. Includes the trade-off between a high level of flood activity (number of floods, duration of floods and area affected) and a high level of impact on human populations (deaths and displacement, and human population density in watershed), and the costs of service protection.

48. As a proportion of all land. The authors examine also the opportunities for service enhancement through landscape restoration.

49. The authors used expert opinion to estimate possible land transformation within the next 5 years. This identified negative and positive changes to service provision.

50. Based on stakeholder preference.

51. The inclusion of stakeholders in the ranking process addresses to a degree the demand for services and/or the value of services to beneficiaries. This is an explicit incorporation of beneficiaries in the process.

52. Based on land management and stakeholder perception.

53. The authors compared the ecosystem-service values to the cost of conserving the natural habitat that underlies their provision. The opportunity cost was calculated as the expected agricultural value of each forested parcel of land.

54. To estimate the economic value of bushmeat the authors used the local market price of a kilo of beef since domestic meat is a possible substitute for bushmeat. This approach implicitly recognises alternatives to service provision.

55. Value for new pharmaceutical products.

56. The authors assumed imminent deforestation outside of core protected areas.

57. Net annual rate of atmospheric carbon added to existing biomass carbon pools (measured using a proxy).

58. The authors’ maximized service provision for a given ecoregion area constraint using optimization methods. Incorporating the issue of area constraints addresses costs, and the maximization goal gets somewhat at demand.

59. Annual production of livestock from grazing on unimproved natural pastures (expressed as tons of meat).

60. Beneficiaries were at the point of production only (where economic benefits are realized). The authors identified production peaks of water provision and grassland production in densely populated biodiversity hotspots, indirectly addressing the issue of spatial variability in demand.

61. Water availability and water use.

62. Only the key points are captured here, see the publication for full details.

63. Scenario analyses explore implications of possible future landscape changes.

64. Estimated through soil loss. Regions with lower potential soil loss were a priority, which implicitly recognises the importance of threats.

65. Water-supply function and flow regulation (mean annual catchment runoff and mean annual groundwater recharge).

66. Identified beneficiaries in the biome through a literature review and expert consultation.

67. Mean carrying capacity of the land incorporating climate, soil type and vegetation.

68. Areas that tourists can see within a 10 km buffer surrounding the major tourist driving routes (see Reyers *et al.* ^55^).

69. Landslide, flood and drought prevention functions.

70. Landslide prevention considered in terms of landslide hazard; the more hazardous an area the more important it is to keep forest in place (an alternative perception of ‘demand’). Drought and flood prevention reflects water retention capability of forest.

71. Estimated using the proximity to settlements and roads (measures of access for deforestation), and distribution of the number of commercial species of trees (a measure of forest desirability for logging).

72. Carrying capacities for domestic stock expressed as the number of hectares required per large stock unit (hectares values were determined for pristine examples of habitat types).

73. The authors compared the potential delivery of ecosystem services from ‘pristine’ locations to that provided by degraded locations, estimating how landscape degradation may diminish the capacity of locations to provide a given service (an indirect assessment of threat).

74. The authors mapped areas vulnerable to erosion and classified them as high, medium and low erosion hazard. Habitat types provide erosion control where there is a high threat of erosion owing to factors such as topography, rainfall and soil (indirectly addressing the issue of threat).

75. Millions of cubic meters of groundwater recharge per 1-km ^2^ grid cell.

76. A related study by Wendland *et al.* ^18^ included costs, threats and demand, but it is unclear if these are included in the measure of hydrological importance used in Rogers *et al.* ^31^.

77. Provision of drinking water to downstream users and irrigation for rice paddies.

78. The authors examined the threats to the biological value of key biodiversity areas (KBAs) based on a ‘human pressure index’ calculated from measures of human population density, road density, fire prevalence and agricultural suitability. They did not directly examine threats to ecosystem-service provision, but did this indirectly by looking at threats to the protection of KBAs, which were ranked based on their hydrological service value.

79. The carbon density of living biomass.

80. The number (and type) of services is a little ambiguous; it appears to be between 9 and 13 depending on the analysis. The authors also conducted analyses at three different spatial scales.

81. Ecosystem service values were expressed in dollar values of land units based on land cover and the services provided by particular land covers.

82. The authors deal with threat(s) to service provision indirectly by modelling the change in ecosystem service value with two alternative development scenarios.

83. The authors calculated the ecosystem-service values ($ value) for 17 different services and recognised variation in the spatial dependencies of services.

84. The authors assessed the vulnerability of biodiversity (‘threat’) and then determined the ecosystem-service value captured in biodiversity templates where low vulnerability is a priority and high vulnerability is a priority.

85. The authors calculated water availability (total and per person) and mapped supply and demand ratios.

86. Water availability per person was referenced against an accepted minimum target (1000 m ^3^) set by the United Nations (hence, this target represents ‘demand’). The authors also calculated the percentage of the population with access to improved water and improved sanitation, and under five mortality per 1000 births.

87. The percentage contribution of carbohydrate and protein-supplying crops to total dietary intake.

88. Service provision is compared to recommended minimum daily intake (2100 kcal per person) and minimum daily intake of protein.

89. Lost agricultural production.

90. Opportunity costs for agriculture and stock.

91. The authors did not calculate water quantity, but used a proxy for the supply of sediment-free water based on population data, land cover and water flow direction.

92. The authors measured downstream users through the downstream populations’ need for quality drinking water, downstream area of irrigated rice fields, and downstream area of mangroves.

## Components of spatial prioritization

The following are key elements to any conservation prioritization problem: biodiversity features [assets] that need protection (e.g., species or habitats); processes that threaten these features (e.g., habitat loss); a set of actions that may be effective at abating the threats (e.g., manage invasive species); and financial information specifying the cost of implementing each action, and the available conservation budget ^11^. ES prioritization shares these elements; that is, identifying ecosystem features that supply services, threats to service provision, potential actions to ensure future supply of services, and the costs of these actions. Yet, prioritization of services requires at least the following additional considerations: the availability of alternative means of providing benefits supplied by services; the capacity of an ES to meet human demands; and scale of, and site dependency in, the delivery of services.

While each of these factors may contribute to the economic valuation of an ES (i.e., captured by a metric such as dollar value) such complete and site-specific economic values are rare. Studies that estimate the financial value of ES facilitate the appreciation of services in widely understood terms, but this approach has well recognised limitations including the fact that financial values under-represent benefits to the poor as they have less capacity to pay than rich people ^21– 23^. Therefore, it is important to explore alternative approaches to identifying spatial priorities for ES management that circumvent some of the limitations of using financial values.

### Supply/benefits of ecosystem services

Quantifying the benefits of protecting the supply of ES is generally most appropriately assessed in terms of the difference between protecting supply and not protecting supply. The advantages of protecting ES supply may be represented as benefits expressed in dollar values or avoided ecosystem damage (e.g., prioritizing locations with high soil erosion potential, but where vegetation cover ensures soil retention; ^24^), or through quantifying the supply of services, often in biophysical units. The latter is the most common approach in broad-scale prioritization studies ( Table 1). Biophysical quantities can include, for example, the amount of carbon stored in particular ecosystem types, water availability or supply, or fodder production. However, it is crucial to address also the issue of the level of biophysical quantity demanded by service beneficiaries. We refer to the level of human need for a service as ‘demand’, but recognise that this level changes with context and differs from the economic perspective of demand as the amount of a good or service that can be purchased at a given price.

Simply increasing the quantity of a given service may/may not be appropriate depending on human need. It could also divert funds from more necessary actions because if the quantities of certain ES are adequate and not under threat, investment in the protection of these services could be a lower priority compared to services currently unable to meet human needs (see ‘Target setting and the capacity to meet demand’). Luck *et al.* ^15^ explicitly addressed this issue by prioritizing locations for managing ES based on the human need for the services of water provision and flood mitigation. This directly links the quantity of service provided with the needs of beneficiaries and better identifies where needs are not being met.

The benefits of managing for ES vary across space and time, reflecting, for example, variation in human need and the capacity to pay for human-derived alternatives. This spatio-temporal variation is decidedly complex, influenced by factors such as the type of service being considered, market fluctuations, and the changing needs of beneficiaries. This dynamism magnifies the complexity of ES prioritization beyond that of biodiversity prioritization. For example, Wilson *et al.* ^11^ note that the benefit-protection function in conservation planning is asymptotic in that benefit accumulation is less and less with the protection of more land. While the same is true for some ES ^25^, the shape of the curve will vary over time and space with beneficiary demand driven by, among other things, markets and changing needs. Moreover, owing to global markets, it can be extremely difficult to identify who benefits from a given service. It is less problematic to focus on the immediate beneficiaries of service provision (e.g., growers benefiting from crop pollination) rather than also considering those individuals that benefit from the products of services (e.g., consumers of crop commodities; ^26^). In some cases, it may be sufficient to recognise simply that the benefits from the provision of a particular service are globally widespread and diffuse (e.g., carbon storage).

### Threats to service provision

Conservation planners may quantify threatening processes that increase the risk of biodiversity loss ^27^ and a similar focus on threats to ES provision is an appropriate way to incorporate threats into service prioritization. It is also important to recognise the fundamental difference between the *vulnerability* of an ES to threat(s), and the *level* of threat a particular service is under. Some services may be particularly vulnerable to threats (e.g., crop pollination reliant on a single pollinator species), but not currently threatened, whereas other services may be resilient to a range of threats, but at risk of decline owing to the magnitude of threat(s).

Despite its importance, few ES prioritization schemes to date have explicitly incorporated threats ( Table 1). Egoh *et al.* ^3^ documented biophysical quantities of ES provided by intact and degraded vegetation, which implicitly addresses threat to service provision through landscape degradation. Others examined changes in quantities or dollar values of services through modelling alternative future land-use scenarios, recognising that some scenarios (e.g., extensive development) represent a greater threat to service provision than others ^28– 30^. A more explicit approach to incorporating threats is to document the likelihood of decline or loss of service-providing ecosystems through, for example, human development or habitat loss ^18, 31^.

Addressing threats to ES is most important when service provision is not substitutable across space (i.e., site dependency is high because the service must be provided in a specific location; e.g., storm protection), there are no human-derived alternatives to service provision or these alternatives are expensive relative to the capacity of local communities to pay for the alternatives, or ecosystem changes are irreversible (e.g., species extinction).

### Costs of actions to manage services

Conservation planners list a variety of costs that should be considered when assessing options for protecting biodiversity ^32^. These range from acquisition costs (e.g., purchasing land for conservation) and management costs (e.g., maintaining conservation areas), through to social costs (e.g., the number of people displaced from conservation areas; ^11, 33^). Costs will vary across space and must be linked to actions to improve planning relevance ^9^. For example, if the action required is land acquisition then a relevant cost is land price; if the action is management of a conservation area then a relevant cost would be the salaries of conservation managers.

The management of ES attracts similar costs dependent on the type of action required to protect the service. Indeed, some ES prioritization schemes incorporate opportunity costs in a similar way to biodiversity prioritization, recognising that managing ecosystems for service provision can yield the same opportunity costs as protecting ecosystems for biodiversity (e.g., when an area cannot be used for production ^3, 18, 34^; Table 1). Costs may also be incorporated through the use of proxies for resource and maintenance expenses (see ‘An example of spatial prioritization’).

It is important to identify the assignation of costs (who pays) and benefits in both biodiversity conservation and ES prioritization ^35^. For example, designation of a conservation area yields benefits that are primarily public, notwithstanding, for example, income generated from nature tourism, but sometimes at a cost to private interests (e.g., opportunity cost of lost revenue from production). Managing an area for the delivery of ES can yield relatively greater private benefits, particularly for service beneficiaries, with costs borne by both public and other private interests. For example, a forest designated for timber harvest will yield financial benefits to logging companies at a cost to the public (e.g., through lost carbon storage) and other private interests (e.g., those interested in using the forest for ecotourism). Ensuring greater equity in the distribution of benefits and costs from services provided by public or private assets may be achieved through various mechanisms such as government regulation, self-regulation (enforced by societal norms), or market approaches like cap and trade or payments for ES ^36, 37^. Yet, the appropriateness of a particular mechanism depends on the characteristics of the service being targeted (e.g., who generates the service, management jurisdiction, and provider–beneficiary spatio-temporal dynamics; see Kinzig *et al.* ^37^).

### Availability of alternatives to service provision

The availability of human-derived alternatives to the provision of ES is a vital consideration in service prioritization. These alternatives can include, for example, a water filtration plant to cover the filtration services of wetlands or pesticides to cover biological control. The availability of alternatives and the capacity of relevant human communities to pay for these alternatives can influence the treatment of other factors such as benefits, threats, actions and costs. For example, managing a particular service may be given lower priority if human-derived alternatives are readily available and affordable, although the associated costs of these alternatives must be considered also (e.g., the health costs of increasing pesticide use). Only a few studies that attempt ES prioritization address the issue of availability of alternatives ( Table 1). As part of the prioritization process, the availability and cost of alternatives should be considered simultaneously with the list of potential actions for service protection or enhancing service provision.

### Target setting and the capacity to meet demand

Setting targets is common in conservation planning and can be a requirement for assessing the capacity of selection procedures to meet conservation objectives ^38^. In most cases, setting a target is equivalent to meeting a baseline threshold. Target setting in ES prioritization is rare and has, to the best of our knowledge, only occurred in four published studies ^3, 4, 39, 40^( Table 1). For example, Chan *et al.* ^39^ set a baseline target (assumed minimum requirement) of 12 days of outdoor recreation per person per year and determined the space required to provide that level of service from data on park visitation. Chan *et al.* ^39^ also stipulated that targets had to be met in different stratification zones within the study area, which accounted somewhat for the site dependency of service production and variability in the spatial distribution of beneficiary needs.

While target setting is one approach to assessing the capacity of ecosystems to meet the demands of beneficiaries, provision–demand relations have been variously dealt with in the literature ( Table 1). For example, some studies included data on water use when calculating water provision capacity [e.g., ^15, 26^], while others measured downstream need for water of a given quality through the calculation of population densities and areas of irrigated rice and mangroves ^18^. Van Jaarsveld *et al.* ^41^ calculated water and food provision relative to accepted minimum standards for human consumption. The need and approach to calculating demand for service provision will vary depending on the service of interest. For example, it is generally considered unnecessary to calculate spatially explicit demand for carbon storage because this service benefits the global community and demand is not spatially variable.

### Site dependency and scale

Site dependency in the provision of an ES reflects the level of need for a particular service to be provided in a particular location in order to deliver benefits to a given set of beneficiaries. This can be interpreted also in the context of the scale of service provision (e.g., local to global). For example, storm protection from mangroves has high site dependency in provision – mangrove forests must occur in locations where local communities are threatened by storm activity. This should not be confused with the substitutability of the service; that is, whether human-derived alternatives (e.g., sea walls) or other coastal vegetation types can provide a similar service. In contrast, global climate regulation through ecosystems storing carbon has lower site dependency in provision because it does not have to occur at a particular location (i.e., there are various options for managing ecosystems to store carbon). However, there is still some level of site preference because certain ecosystems (e.g., rainforests) store more carbon than others. Site dependency and scale varies also in the use of the service. For example, the beneficiaries of biological control in agro-ecosystems generally occur at the local to regional scale, if the emphasis is on growers, whereas the beneficiaries of climate regulation occur at the global scale.

Variation in the site dependency and scale of the provision and use of ES has major implications for the valuation of services, which must consider spatially explicit and scale-dependent relationships in production–consumption flows ^42^. Such relationships also have important implications for prioritization strategies. High site dependency could result in certain locations that generate that service being classified as irreplaceable. For example, Bohensky *et al.* ^43^ identified irreplaceable land units for food and water provision to meet pre-determined targets of caloric intake for a given population. When services have lower levels of site dependency in production there is greater flexibility in site selection during the prioritization process (all else being equal).

## An example of spatial prioritization

**Figure 1.**
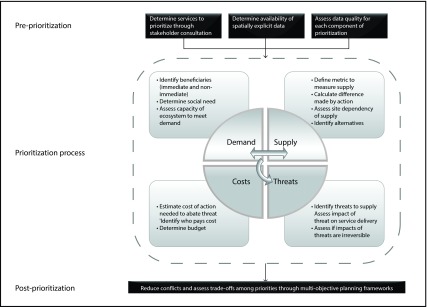
Key aspects for consideration in ecosystem-service prioritization.

The relationships among the various components of our conceptual framework for spatial prioritization of ES are presented in Figure 1. We illustrate our approach in this section using a worked example based on data published in Luck *et al.* ^15^ focussing, for the sake of simplicity, on a single ES: water provision.

The global analysis of Luck *et al.* ^15^ identified watersheds that are a priority for protecting particular ES. The first step in the analysis was to quantify the benefits and supply of the service. The benefits of protecting the supply of potable water was measured through human population density in each watershed; that is, there were greater benefits to protecting supply in watersheds with higher population density compared to those with lower density. Water supply was measured using a global hydrological model, and ‘water-production efficiency’ was calculated for each watershed by dividing supply in each watershed with watershed area.

The costs of actions to manage water provision were represented using a proxy for resource (e.g., land acquisition and infrastructure) and maintenance (e.g., labour) costs. This proxy incorporated data on total income in the watershed (per capita gross national income), population size and watershed area. Resource costs were assumed to scale positively with per-capita wealth and population density (assuming that land and infrastructure prices are generally higher where population density is higher), while maintenance costs were assumed to also scale positively with per-capita wealth. Finally, the cost-effectiveness of protecting the service in each watershed was calculated by dividing human population density and water supply (benefits) by the cost.

The capacity to meet demand was measured using values for water supply and water withdrawals in each watershed. It also considered regional water deficits (withdrawals > supply) and the proportion of total supply that remained once demands were met, adjusting the watershed-level capacity measure downwards proportional to the need to move water to regions (within a watershed) where supply did not meet demand. It was assumed that managing the service of water provision was most important in watersheds where supply barely meets or is short of demand, and less important when supply greatly exceeded demand.

To estimate threat to water provision, expected vegetation cover in each watershed was used, recognising the link between vegetation and water provision, filtration and the maintenance of water quality (although this link is decidedly complex; see Luck *et al.* ^15^ for details). Vegetation cover and type in a watershed may be indicative of the capacity of the watershed to provide potable water naturally, and change in vegetation cover can be considered a proxy for threat to water provision. To quantify this threat, the following data were used: the proportion of each watershed covered in tree, shrub and herbaceous vegetation; the annual rate of change in vegetation cover (over a proceeding 5-year period); the time span over which change in cover would be predicted (e.g., 20 years); and the proportion of the watershed that was protected (assuming vegetation in protected areas could not be cleared). Watersheds with mid-range values of vegetation cover, rates of vegetation loss and/or area protected were considered priorities for water provision management, because, for example, watersheds with low cover and high rates of loss would require large investments in ES management relative to return, whereas watersheds with high cover and low rates of loss are under less threat to the disruption of the service.

The final consideration in spatial prioritization is the availability of alternatives to the provision of the service via ecosystems. Improvements in the supply of potable water may be made through the construction of dams and building of filtration plants, for example, rather than ecosystem management. The availability of these alternatives is often a function of the capacity of local communities to pay for them, in addition to other constraints (e.g., topographic suitability for dam construction). Therefore, Luck *et al.* ^15^ used the gross national income per capita of countries spanning each watershed as an indicator of the capacity of communities reliant on the watershed to pay for alternatives to natural water provision.

The above components were combined into a single index representing the relative importance of each watershed for protecting water supply. This example, and our prioritization framework generally, is appropriate when planning units are large and there are a variety of available options for managing services, and it is difficult to express the components of prioritization precisely. Our framework treats the supply of services, threats and costs in ES prioritization inclusive of beneficiary demand, capacity to meet demand, and availability of alternatives to ES provision.

## Discussion

Our review of current approaches to identifying spatial priorities for managing ES found that the important components of prioritization (benefits, costs, threats, availability of alternative, and capacity to meet demand) were treated in substantially different ways or sometimes omitted completely (although not all components are applicable in every context). Moreover, few studies explicitly addressed the issue of site dependency and scale in the provision of services and/or location of beneficiaries. Accordingly, there is substantial scope for improving ES analyses aimed at identifying spatial priorities for managing services.

If ES benefits were commodities in perfect markets, the price of such benefits would reflect all of the spatial prioritization components identified above. Yet, many ES benefits are not commodities, and for those that are, the associated markets are far from perfect, suffering from numerous market failures including monopsony (single buyers, as in reverse auctions), oligopoly (few sellers, as in many payments-for-ES schemes), externalities and information asymmetries. This means that market prices will not generally reflect all of the components of prioritization appropriately. Stated-preference non-market valuation approaches can be informative in certain settings where markets do not apply, but they are generally of limited utility reflecting the various dimensions of value/social priority ^44, 45^. Accordingly, even where economic valuation data are available, it will still be appropriate for ES prioritization exercises to separately integrate some of the components we identify.

Although we have focussed on the mechanics of prioritization, the following issues must be addressed prior to such analyses: 1) identification of the ES to be included; 2) capacity to access spatially explicit data; and 3) data quality ( Figure 1). Identifying important ES should occur through in-depth consultation among scientists, policy-makers, managers and stakeholders (especially service beneficiaries; see Fisher *et al.* ^46^). For example, if prioritization was required across a particular country, federal management agencies and relevant stakeholders may engage in a process of identifying those services most important to the well-being of the country. ‘Importance’ may be a factor of the total financial value of a service (e.g., agricultural production) and/or the societal need for a service (e.g., provision of potable water) and assessed through appropriate valuation approaches. Hence, a priority list of which services to focus on is required prior to deciding on where to invest in ES management. The selection of different services will influence the approach to spatial prioritization because ES management will have different objectives (e.g., improving timber harvest or maintaining water supply). However, the major steps we outline here will be relevant in most cases.

Application of our approach requires spatially explicit data on where services are produced, benefits, costs and threats. Data on spatially explicit production is usually obtained through mapping the location of ecosystems that provide services (e.g., grasslands that support livestock). Mostly, this involves maps of vegetation types or water sources ( Table 1). Benefits are represented spatially generally via the biophysical quantity of a given service produced by a given location (e.g., carbon stored) and/or its financial value. To represent costs spatially, researchers have used simply the area of the planning unit (e.g., ^39^, for some services) or current land values [e.g., ^34^]. Documenting costs is problematic because of spatio-temporal variation in financial values, which is possibly why some researchers have resorted to more simple rules-of-thumb (e.g., assuming that managing larger areas yields greater costs). Also problematic is spatially explicit measures of threat, which have been represented by, for example, maps of land-use or historical or potential land-cover change and how these relate, spatially, to the location of service provision [e.g., ^30, 47^] ( Table 1).

Finally, the type of data used in prioritization will greatly affect outcomes. For example, Anderson *et al.* ^2^ demonstrated that variation in the resolution (≈ grain size) and/or spatial extent of a prioritization analysis influenced the level of congruence between biodiversity and ES priorities. Moreover, data quality may be poor for certain services and certain components of prioritization. For example, crude proxies or indicators may be required for services for which it is difficult to obtain accurate, spatially explicit measures of supply and demand (e.g., flood mitigation; see Holland *et al.* ^48^). Our framework, which promotes the use also of data on threats, costs, alternatives and site dependency, may help to alleviate this issue because prioritization could be based just on those components for which data quality is acceptable.

The most appropriate metric to represent the supply of the service will be context dependent, but the use of biophysical quantities will be suitable in most cases. For example, if the service is storm protection then a suitable metric may be the area of mangroves that needs to be maintained to deliver a given level of protection [e.g., ^25^]. ES supply should be assessed relative to the demand for the service, which can be measured using a target-based approach, through current or projected use of the service or its products, through demonstrated need for the service (e.g., historical impacts of storms) or through meeting an accepted minimum standard (e.g., acceptable losses due to storm damage). Quantifying demand for a service requires the implicit or, preferably, explicit identification of beneficiaries, which may be immediate beneficiaries (e.g., residents of coastal villages threatened by storms) and/or ‘non-immediate’ beneficiaries (e.g., consumers of goods produced by the villages).

The application of prioritization frameworks generally involves multiple services across many planning units and priorities for different services are not necessarily congruent ^2^. This requires an analysis of trade-offs between services in managing land/sea-space for service provision ^5^. Moilanen *et al.* ^49^ addressed this issue using a multi-objective prioritization approach for biodiversity and ES based on the conservation planning software Zonation. The authors argued that regional variation in land-use priorities meant that spatial separation of land uses may reduce management conflicts. Moreover, areas that are a priority for multiple ES could be used in trade-offs assuming the areas were not critical priorities for every service.

In multi-objective prioritization frameworks that consider spatial separation of ES management or trade-offs in land-use priorities, it is vital to address site-dependency and scale of service provision and location of beneficiaries. As we argue above, there is little flexibility in managing for the provision of services that are delivered locally to *in situ* beneficiaries (e.g., flood mitigation). Avoiding inappropriate management decisions and trade-offs rests entirely on taking a comprehensive approach to identifying priorities. Here we describe the major factors that must be considered in ES prioritization and argue that addressing as many of these factors as possible will improve the outcomes of multi-objective prioritization frameworks that aim to promote human well-being through the protection of services.

Developing comprehensive methods for identifying ES priorities is much more than just an academic exercise. Governments and NGOs across the world are increasingly including the protection of ES into their policy directives. For example, the governments of China, Costa Rica and Mexico pay landholders that engage in management that protects the supply of hydrological services ^50– 52^. Vital to this process is identifying locations that offer the greatest return on investment. This requires a systematic and thorough approach to identifying spatial priorities for protecting ES.
