# Supplementary material for: Identifying spatial priorities for protecting ecosystem services
Source: F1000Res. 2012 Sep 27;1:17. [Version 1] doi: 10.12688/f1000research.1-17.v1 (PMC3814920; doi:10.12688/f1000research.1-17.v1)
Supplement: Peer review history

# Peer review: Referee response for version 1

**Published:** 2012-10-04

**Authors:** David Norton

**Affiliations:** 1 College of Engineering, University of Canterbury, Christchurch, New Zealand

This is an interesting article that provides a conceptual framework for prioritizing areas for protecting ecosystem services (ES) such as water provision.

The article explores similarities and differences between ES prioritization and biodiversity prioritization, and highlights several key components that need to be considered in prioritizing areas for protecting ES. Two key components are the need to consider the costs of protecting areas and the importance of considering human-derived alternatives. The conceptual framework is then applied to a case study based on water provision.

I have read this submission. I believe that I have an appropriate level of expertise to confirm that it is of an acceptable scientific standard.

# Peer review: Referee response for version 1

**Published:** 2012-09-29

**Authors:** Elena Bennett

**Affiliations:** 1 Natural Resource Sciences and the McGill School of the Environment, McGill University, Ste Anne-de-Bellevue, Canada

This work presents an interesting conceptual framework that researchers as well as governments or other stakeholders could use to identify priority areas for protecting ecosystem services.

The authors suggest the following key elements: (1) determine the services of interest, (2) quantify the demand for the service(s) and capacity of the ecosystem to meet demand through supply of the service (3) identify the threats and level of threat, and (4) estimate the cost of protecting the service as well as any alternative means of providing the service. The authors then review the literature in which priorities have been assessed and identify which steps have been taken and which not in each study or effort. I hope this paper will increase our attention to key aspects of ES assessment that are often ignored, especially the interaction among all of these key factors.

I have read this submission. I believe that I have an appropriate level of expertise to confirm that it is of an acceptable scientific standard.
